# Fish—To Eat or Not to Eat? A Mixed-Methods Investigation of the Conundrum of Fish Consumption in the Context of Marine Pollution in Indonesia

**DOI:** 10.3390/ijerph20085582

**Published:** 2023-04-19

**Authors:** Oyedolapo A. Anyanwu, Sara C. Folta, Fang Fang Zhang, Kenneth Chui, Virginia R. Chomitz, Martha I. Kartasurya, Elena N. Naumova

**Affiliations:** 1 Friedman School of Nutrition Science and Policy, Tufts University, 150 Harrison Ave, Boston, MA 02111, USA; 2 Public Health & Community Medicine, School of Medicine, Tufts University, 136 Harrison Ave, Boston, MA 02111, USA; 3 Department of Public Health Nutrition, Diponegoro University, Semarang 50275, Jawa Tengah, Indonesia; mkartasurya64@gmail.com

**Keywords:** fish consumption, marine pollution, awareness, food security, public health

## Abstract

**Background:** The Indonesian government faces a dilemma of promoting fish consumption for its health benefits and to ease food insecurity, while at the same time seeking effective approaches to reduce the high levels of marine pollution. However, the factors associated with fish consumption in the face of persistent high levels of marine pollution are not well elucidated in the literature. **Objectives:** This was an explorative study to investigate the sociodemographic factors related to fish consumption and to understand the perspectives of expert informants on marine pollution and its impact on fish quality and availability in Indonesia. **Methods:** We characterized fish consumption among respondents aged 15 years and older in the fifth wave of the Indonesian Family Life Survey (*n* = 31,032), based on their sociodemographic profiles, and developed multinomial regression models to assess the relationship between respondents’ sociodemographic profiles and quintiles of fish consumption. We also conducted in-depth interviews on fish consumption and marine pollution with key informants from Indonesia (*n* = 27). We then used a convergent mixed-methods design to synthesize the results of both datasets. **Results:** Fish was the most frequently consumed animal-source food reported by survey respondents: 2.8 (±2.6) days/week. More younger respondents (15–19 years) reported relatively lower consumption of fish (9.3% in Q1 versus 5.9% in Q5) compared to respondents 50 years and older (37% in Q1 versus 39.9% in Q5; *p* < 0.01). When classified by region, more respondents from the Java region reported lower consumption of fish (86.5% in Q1 versus 53% in Q5; *p* < 0.01). Key-informants’ perspectives corroborated the survey results by indicating that the younger generation tends not to want to consume fish; informants expanded the survey results by suggesting that fish is scarce in the Java region due to high levels of marine pollution. Informants further implied that there is low awareness about the impact of marine pollution on fish quality among most of the Indonesian population. **Conclusion:** Evidence from both data sources converge on differential preference for fish consumption by age group. Informants’ perspectives also link marine pollution to fish scarcity, which poses a threat to food security among low-income Indonesians and to human health globally. More studies are needed to corroborate our findings and inform policy guidelines to reduce marine pollution while promoting fish consumption in Indonesia.

## 1. Introduction

Fish, an important human health asset, provides >20% of animal protein for 2.6 billion people globally [1]. Fish is also a good source of essential amino acids for various metabolic functions, including cognitive development and cardiovascular health [2,3]. Hence, fish is often considered a healthier source of protein than other animal protein sources like red meat, which has more saturated fat content and could potentially pose a health risk [1,4,5]. As an important complementary food to diets characterized by calorie-dense staples like rice, fish consumption facilitates a healthy and more balanced dietary pattern [3]. Thus, fish and marine products contribute significantly to global food security [6].

Many lower and middle-income countries (LMICs) experience a double burden of malnutrition, where undernutrition co-exists with overweight [7,8]. Researchers have proposed solutions to address concurrent malnutrition issues in these countries [7,9]. Fish consumption is considered one such approach, particularly for coastal populations like those of Indonesia, for whom fish is a major protein source and an important source of revenue [10,11]. Fish is not only important nationally, but also, Indonesia is the second-largest producer of fish in the world [12]. As part of its food security policy, the Indonesian government aims to increase access to nutritious foods, which include higher fish consumption [10,11]. Since its inception in 2000, the Ministry of Marine Affairs has launched the ‘Love Eating Fish’ campaign to create greater awareness about the benefits of consuming fish. Consequently, fish consumption in Indonesia rose from 19 kg/capita/year in 2010 to 54.5 kg/capita/year in 2019 [2,13]. Despite this increase, fish consumption is still lower in Indonesia than in some other Asian countries [5]. Moreover, there is insufficient evidence about fish consumption by sociodemographic factors to assess the levels of intake and identify the specific sub-populations at risk for lower fish consumption [13,14]. Such relationships are worth investigating with both quantitative and qualitative data, using mixed-methods approaches that allow us to better clarify underlying contextual factors than one approach alone [15].

A healthy marine ecosystem is very important for fish availability and quality. Indonesia, the world’s largest archipelagic country, also has one of the highest levels of marine pollution in the world, ranking second after China [16,17]. An important contributor to marine pollution in Indonesia is nutritional transition, a process of rapid shifts in dietary patterns from minimally processed traditional staples to a greater consumption of ultra-processed foods that have high contents of saturated fat, salt and sugar [9,18,19]. These marked changes in diets have been linked to the global epidemic of chronic diseases, with many LMICs, including Indonesia, shouldering the largest burdens of related economic and health costs [20,21]. Undergirding the rapid nutritional transition are economic development and demographic transition, with increasing urbanization and globalization of food-distribution systems [9,22,23]. Multinational supermarkets and fast-food chains have penetrated the food retail outlets in many urban centers, displacing local markets where people typically get fresh produce [22,23,24,25]. These changes have also favored higher consumption of animal-source foods [26]. Against this backdrop, Indonesian oceans and seas are polluted from many sources, including single-use plastics, industrial wastes from industries, run-off from fertilizers and pesticides, oil spillage, and so on [27]. Nevertheless, few studies have investigated the impact of nutritional transitions on the marine environment in Indonesia [28,29]. Bamberger et al. used a business-as-usual model to assess the impact of the nutritional transition on key environmental indicators, such as marine eutrophication in seven emerging countries, including Indonesia, from 2011 to 2030 [29]. They projected that the environmental impact would be highest for Indonesia, with a clear trend for higher consumption of ASFs, vegetable oils, and fats for all the countries [29].

Previous studies on the impact of marine pollution on marine species and humans have revealed a dose-response relationship, whereby the adverse effects begin to show only after a certain period of exposure [30,31]. There are different pathways by which marine pollution can pose health risks. Fish has the capacity to accumulate heavy metals such as mercury and lead [30]. Pollution from ocean plastics can also kill fish and marine species and thereby contribute to food insecurity, especially where seafood is the major source of protein for the population [16,32]. Microplastics ingested by humans through consuming fish or other seafood may cause inflammation and laceration of tissues in the gastro-intestinal tract [16]. Ingestion can also lead to alteration in chromosomes, which can cause infertility, obesity and cancer [33]. Chemicals from plastic debris can be directly toxic: for example, monomers of polystyrene, polyurethane and polycarbonate can be carcinogenic [34,35], or indirectly toxic, through the release of persistent bio-accumulating and toxic substances (PBAs) that may have gathered in the plastics over time [36]. Insecticides and other organic pollutants are also found on plastic wastes in harmful concentrations [37]. These chemicals are bioavailable to fish and other marine animal species and can be transferred to humans, thus increasing the burden of chemical contamination in humans [16,38]. Additionally, microplastics can interact with heavy metals and increase health risks for humans [38,39]. However, all these studies used quantitative approaches and did not address the socio-cultural context of marine pollution in Indonesia [40]. Further, little is known in the literature about the level of awareness among Indonesians concerning marine pollution and its impact on fish availability and quality.

This study therefore sought to broaden the evidence base on fish consumption and marine pollution in Indonesia by synthesizing evidence from two methods of research inquiry, qualitative and quantitative, to understand the challenges related to promoting fish consumption concurrent with high marine pollution levels. The further aim was to generate hypotheses about how awareness of marine pollution may influence fish consumption. The mixed-methods approach allows for triangulation—comparing, corroborating, and extending the evidence from one data source to another, or further investigating areas of divergence [15]. Our findings may help inform policy guidelines and the design of programs that promote sustainable and safe fish consumption for the population while ensuring the health of the marine ecosystem.

## 2. Methods

**Figure 1:**
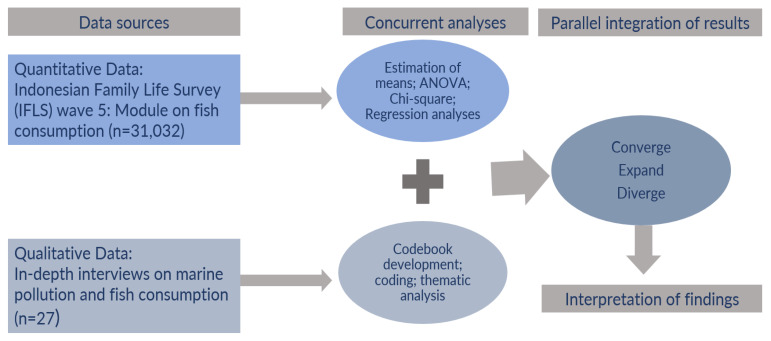
Study Flowchart for the Convergent Mixed-Methods Design.

This study employed a convergent mixed-methods design to investigate factors related to fish consumption in the context of high levels of marine pollution (Figure 1). The convergent mixed methods design allows for the concurrent analyses of quantitative and qualitative data, with the intent of triangulating the findings from both data sources to arrive at a holistic picture of the research topic of interest [15]. Our rationale for combining both data sources was to get a clearer understanding about the contextual factors that influence fish consumption in Indonesia than either data source alone can provide [15]. More specifically, this procedure involves first describing each of the data sources, including the recruitment, location, and study population, as well as the methods employed to analyze each dataset (Figure 1). Next, we present the findings from both analyses in a parallel integrative format [41], and then in the discussion section, we offer our interpretation of these findings and define areas of future research [15].

### 2.1. Description of Data Sources

#### 2.1.1. Quantitative Data

We conducted a cross-sectional assessment of the food consumption module of the fifth wave of the Indonesian Family Life (IFLS 5) dataset based on the Strengthening the Reporting of Observational Studies in Epidemiology (STROBE) cross-sectional reporting guidelines [19,42]. The IFLS is an ongoing, national survey that assesses dietary intake and health indicators of Indonesian adults and children. The IFLS was stratified by province and urban/rural location, and households were randomly selected from these strata based on a sampling frame derived from the Indonesian Bureau of Statistics [43]. A total of 321 enumeration areas (EAs) were randomly selected from 13 provinces, and EAs in urban and smaller provinces were oversampled to facilitate urban-rural and Javanese–non-Javanese comparisons [43]. The ethical approval for IFLS 5 was provided in the United States by the Institutional Review Board (IRB) of the Research and Development Corporation (RAND) and in Indonesia by the IRB of the University of Gadjah Mada [44]. Prior to the start of the survey, each respondent provided a written and signed consent to participate in the study [45]. The IFLS data are publicly available upon request and creation of a free online account with RAND. The fifth wave (IFLS 5), used for this study, was conducted in 2014 and administered to 16,204 households consisting of 50,148 individuals [43]. Only respondents 15 years and older (*n* = 31,032) with complete data on sociodemographic and food-consumption variables were included in the analysis [19].

#### 2.1.2. Dependent Variable

The food-consumption module of IFLS 5 asked about the consumption of 17 food items. Most of the items included were iron- and vitamin A–rich foods, which are two micronutrients of public health importance for Indonesia [43]. Respondents were asked whether and how often they had consumed each food item in the past week [43]. The dependent variable of interest was levels of fish consumption in the past week. Respondents’ responses ranged from 0 to 7 days. We assessed mean fish consumption in the past week and compared it to consumption of other ASFs: meat, eggs and dairy. Next, we categorized fish consumption into age- and gender-specific quintiles to assess how each level of fish consumption is associated with respondents’ sociodemographic characteristics [19].

#### 2.1.3. Independent Variables

Sociodemographic information collected during the survey included age, gender, urban-rural location, region, marital status, education level, and involvement in fishery occupation. To assess involvement in fishery, survey respondents were first asked about their involvement in agricultural or non-agricultural business, and those who were involved in agricultural business were asked whether they practiced fishery [45]. We categorized agricultural involvement into three levels: non-farmer, non-fish farmer and fishery occupation. Education is a key demographic variable that has been considered a proxy for socio-economic status and has been found to be associated with many health behaviors [19]. Respondents were asked about their highest level of education, and responses ranged from less than primary to university level [45]. We categorized these into four: primary, secondary, some college, and bachelor’s degree and above, based on the major education-level distribution in the dataset. Anthropometric measures of height and weight were also collected. To assess weight status, each respondent’s weight (kg) and height (cm) were taken, based on the study protocol [43]. Height was recalculated into meters by dividing the cm values by 100, and body mass index (BMI) values were calculated based on the formula kg/m^2^. BMI values were categorized into four levels using cut-off points recommended by the World Health Organization [46] for an Asian population: underweight < 18.5, normal = 18.5 to <23, overweight = 23 to 27.5 and obese >27.5 [47].

### 2.2. Statistical Analyses

We compared the mean consumption of fish to the mean consumption of other animal-source foods in the past week, stratified by the respondents’ age group. We conducted ANOVA tests (for continuous variables) and chi-square tests (for categorical variables) to assess associations between each quintile of fish consumption and sociodemographic variables (age, gender, urban-rural location, region, fishery occupation, educational level, and weight status). We developed multinomial logistic regression models to assess the magnitude and direction of association between the lowest and highest quintiles of fish consumption, and respondents’ sociodemographic factors: age, gender, place of residence, region, education level, marital status, and fishery occupation. The full model included all the sociodemographic variables and additionally adjusted for consumption of other ASFs and weight status. We plotted multi-panel probability bar charts from the full model to visualize the pattern of fish consumption at each quintile based on the category of each sociodemographic variable. Statistical significance was determined with a *p*-value of 0.05 or lower. Regression analyses were performed using Stata version 17 (StataCorp LLC 2021, College Station, TX, USA), and accounted for complex survey design and attrition. The Stata software algorithm returns regression coefficients as relative probabilities, called relative risk ratios (RRR) rather than as odds ratios (StataCorp 2021 Stata: Release 17 Base reference manual pp. 1605–1606). In the regression output table (Supplementary Table S1), we retained these coefficients as RRR, but in the results and discussion sections we interpreted them as odds or likelihood of fish consumption at the highest quintile relative to lowest.

#### Statistical Models

Multinomial logistic regression models were developed, based on the assumptions for logit models: categorical outcome variable, independent observations, little or no collinearity among independent variables, and large sample size [48]. Reference categories for each categorical variable were chosen based on whether the number of observations for the category was large enough to have statistical power for comparisons with the other categories of the variable.

### 2.3. Qualitative Data

The qualitative data was extracted from a larger qualitative study based on the socioecological model (SEM) [49]. We sought the perspectives of key-informants on diets and health outcomes, particularly risk factors for cardiovascular disease (CVD) in Indonesia [23,40]. The research guidelines of the Consolidated Criteria for Reporting Qualitative (COREQ) were used to report this study [50]. Research questions addressed the importance of fish to the diets of Indonesians; factors related to marine pollution; the impact of marine pollution on fish quality and availability, and the general awareness of the Indonesian population about the impact of marine pollution on fish quality. The study team comprised researchers from Tufts University, Boston, MA and Universitas Diponegoro, Semarang, Indonesia (ENN, VRC, FFZ, KC, MIK and SCF) and one PhD student (OAA), who coordinated the study as part of her doctoral thesis. The team was multi-disciplinary, with many years of research experience in the fields of nutrition, epidemiology, public health, and environmental health. Details of roles and the contributions of each team member to the study have been previously documented [23,40].

We conducted in-depth interviews via Zoom with a purposive sample of key informants from across Indonesia (*n* = 27), between April and June 2021, using a snowball recruitment strategy. The criteria for recruitment have been previously published [23,40]. The major requirements were English-language proficiency and appropriate expertise to respond to the research questions. Informants were healthcare providers, public health nutrition researchers, or environmental researchers. The sample size was deemed adequate based on the range of perspectives and saturation on research topics [51,52]. Saturation was determined when no new information or perspective came to light that had not been previously expressed [51]. The institutional review boards (IRB) of Tufts University and the Universitas Diponegoro (UNDIP) Indonesia approved the study. A semi-structured interview-question guide was developed based on the SEM framework described above. Sections of the guide addressed environmental factors that affect dietary behaviors and health outcomes (Appendix A). The question guide was reviewed by study collaborators from Indonesia to make sure the questions were culturally relevant. Next, we pilot-tested the interview guide with two Indonesians who had similar demographic characteristics and expertise with the key-informants recruited [23,40]. Interview sessions were recorded, and the duration of each session was 45 to 60 min. To validate the perceptions of informants, the interviewer provided a summary of the main points addressed at the end of each session [23,40].

#### Data Analysis

We transcribed the audio files from the in-depth interviews verbatim and iteratively developed codebooks from the transcripts based on a deductive approach [52]. For intercoder-reliability process, three transcripts were randomly selected and independently coded by two research team members [23,40]. There were no major differences in coding other than the necessity to define the inclusion and exclusion criteria more clearly for some codes. After clarifying the differences, the overall intercoder reliability score was >90% agreement and at least 0.75 on the kappa coefficient for all codes [23,40]. We used the NVivo software (Version 12 QSR International; 2018) for the analysis. We reviewed and refined emerging themes iteratively, using a primarily deductive approach, even though some themes emerged inductively [52].

### 2.4. Data Integration Plan

To integrate the results of the analyses of the fish consumption survey data in IFLS with the analyses of the in-depth interview data, we utilized a parallel integration approach for a convergent mixed-method design [15,41]. This procedure involved comparing the findings from both datasets in a side-by-side narrative format to determine in what ways the results converged, expanded upon or diverged from one another [15,41].

## 3. Results

### 3.1. Sociodemographic Characteristics of IFLS 5 Survey Respondents and In-Depth Interview Informants

Table 1 shows the demographic characteristics of IFLS 5 respondents stratified by age group. Overall, about 54% were female, but among respondents 15 to 19 years, the majority were male (54%). The mean age was 42.4 years (±15.8). Breakdown by place of residence was approximately evenly distributed by urban and rural location. About 73% were from the Java region and 16% from the Sumatra region, followed by N. Tenggara (5%), Kalimantan (3%) and Sulawesi (3%). Educationally, 44.6% had primary schooling or less, 44.4% had a secondary level, about 3% had some college education, and 8% had a bachelor’s degree or more. Occupationally, about 60% were not involved in any agricultural occupation, 38% were involved in agriculture, but not fishery, and 2% were involved in fishery. Most of the respondents were of normal weight (39.6%), while the prevalence of overweight and obese respondents was 30.9% and 17.4%, respectively. Table 2 shows the demographic characteristics of key-informants with expertise to address fish consumption and marine pollution in Indonesia. Women made up about 70% of the sample. Javanese was the most common ethnicity (52%), and the mean age was 46 years (±11.9). About 48% of the informants held a PhD, 22% had a professional medical or nutritional qualification, 18% held a master’s degree, and the remaining 11% had a bachelor’s degree.

### 3.2. Themes on Marine Pollution and Fish Quality and Availability

Table 3 summarizes the major themes that emerged from the in-depth interviews. Key informants’ perspectives evoked three major themes related to marine pollution and fish quality and availability in Indonesia: the importance attached to fish consumption among Indonesians; factors related to marine pollution, and the impact on fish quality and availability; and the population’s awareness of marine pollution. The three themes are presented alongside the results from the survey on fish consumption among respondents in IFLS 5.

### 3.3. Consumption of Animal-Source Foods (ASFs)

Table 4 shows the mean consumption of ASFs in the past week by IFLS 5 survey respondents stratified by age group. Overall, fish was the most frequently consumed ASF (2.8 days/week), with respondents 50 years and older reporting the highest level (2.9 days/week) and respondents 15 to 19 years reporting the lowest level (2.4 days/week) of consumption. Eggs were the second most frequently consumed ASF reported in the past week (2.4 days/week), with the highest consumption among respondents 20 to 49 years (2.6 days/week) and least consumption among those 50 years and older (2.2 days/week). Consumption of meat (chicken, beef, pork) was reported 1.5 days/week on average among all respondents, with the highest consumption among respondents 15 to 19 years (1.6 days/week) compared to respondents 20 to 49 (1.5 days/week) and older (1.3 days/week). Dairy was the least consumed ASF among all respondents (1.1 days/week).

Key informants related their views about consumption of fish and other ASFs. They stressed the importance of fish to the nutrition of Indonesians: It is considered an affordable source of protein for many people, and cheaper than other animal protein sources like beef or chicken. It is considered good for brain and heart health because it is rich in omega 3 and minerals and builds the body’s defense mechanisms. However, informants noted that some sub-populations, particularly younger people, tend not to like fish.

*The good source of protein is from fish … we can have it in an eggs or meat, but meat is very expensive... Fish is very cheap and available for the whole family, for the whole community and they know it*.

(65-year-old female, a healthcare provider).

*Oh, I think fish is very important in our diet because from my patient, I know that some of them they really know why they have to eat fish. Some of them say that fish contains good fat, it differs from other animal protein like red meat so they just choose fish compared to meat because it can impact their health. Some of them say that if they eat more fish other than red meat … they will decrease the cardiovascular risk*.

(38-year-old female, a healthcare provider).

*Many, many tourists, many people from many countries came to Indonesia to eat their seafoods, but their own people do not like this. Maybe, they want to eat, but very little. The older people want to eat seafood, but the millennials, the 19s, the 20s doesn’t like these food types*.

(29-year-old male, a healthcare provider).

### 3.4. Sociodemographic Factors Related to Fish Consumption and Marine Pollution

Table 5 shows the bivariate distribution of quintiles of fish consumed, based on respondents’ sociodemographic characteristics. Younger respondents (9.3% in Q1 versus 5.9% in Q5, *p* > 0.01) and living in the Java region (86.5% in Q1 versus 53% in Q5, *p* > 0.01) were significantly associated with lower consumption of fish. Also, urban dwellers were more likely than rural dwellers to be classified in the lowest quintile of fish consumption (49.2% in Q1 versus 41.9% in Q5 and 50.8% in Q1 versus 58.1% in Q5 respectively; *p* > 0.01). Further, respondents classified in the highest quintile of fish consumption were older, more likely to be rural dwellers, of the male gender, married and overweight or obese. They were also more likely to have a bachelor’s degree or higher and to be involved in a farming or fishing (Table 5).

**Figure 2:**
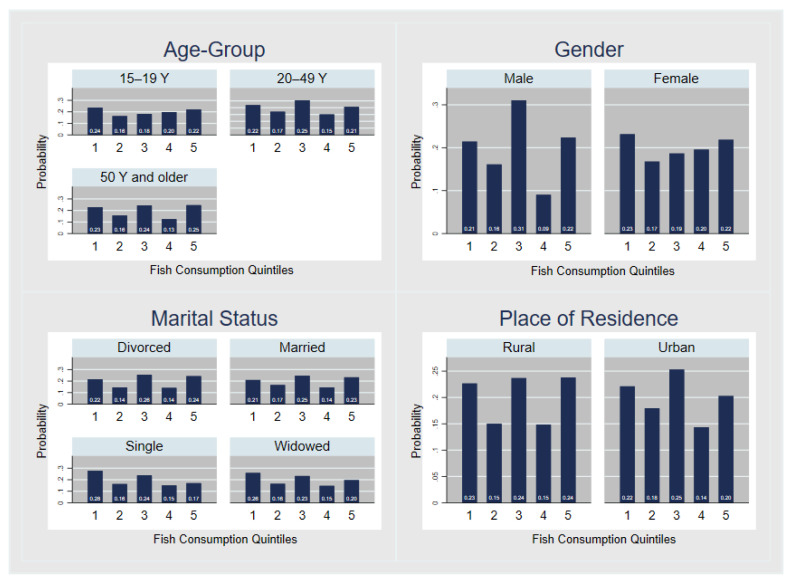
Bar charts showing the predicted probabilities of being in each fish consumption quintile by age-group, gender, marital status, and place of residence.

**Figure 3:**
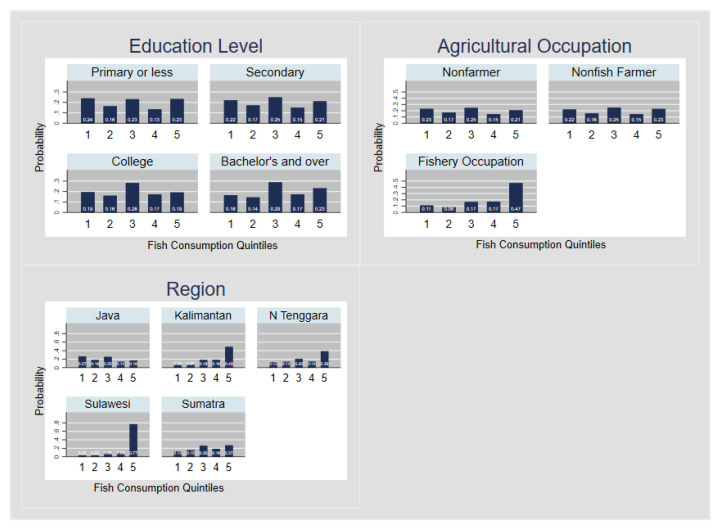
Bar charts showing the predicted probabilities of being in each fish consumption quintile by educational level, involvement in fishery, and region.

Results of the multinomial regression analysis are tabulated in Supplementary Table S1, and Figure 2 and Figure 3 show multi-panel bar charts of predicted probabilities of being in each quintile of fish consumption. Compared to other age-groups, respondents 50 years and above had 18% higher odds of fish consumption at quintile 5 relative to 1 (RRR = 1.18; 95% CI 1.05, 1.33; *p*-trend < 0.01). Distribution by gender revealed no significant difference between male and female respondents in being classified at quintile 5 relative to 1. Classification by place of residence showed that urban dwellers compared to rural dwellers were 15% less likely to be in quintile 5 relative to 1 (RRR = 0.85; 95% CI 0.76, 0.94; *p*-trend < 0.01). When compared with respondents with primary education or less, those with a bachelor’s level and over had 84% increased odds of fish consumption at quintile 3, relative to 1 and 49% higher odds of being in quintile 5 relative to 1 (RRR = 1.84; 95% CI 1.56, 2.18 and 1.49; 95% CI 1.25, 1.78 respectively; *p*-trend < 0.01). Compared to non-farmers, respondents involved in fishery were 5.65 times more likely to be in quintile 5 relative to 1 (RRR = 5.65; 95% CI 4.10, 7.79; *p*-trend < 0.01). Classification by region showed that compared to living in Java, living in a non-Javanese region was associated with higher odds of fish consumption at quintile 5 relative to 1 (Figure 3, Supplementary Table S1).

Informants evoked the perspective that fish is scarce in some regions of Indonesia, particularly in the Java region, and alluded to high rates of pollution as a contributory factor:

*I don’t know if your focus is on Java, practically now, it’s quite hard to have a big harvest of seafood on the north coast of Java ... because of the quality of the ecosystem, it’s getting lower. The quality is reduced and the possibility to have a big catch is also reduced*.

(59-year-old male, an environmental researcher).

*I think in Java, we consume low quality of fish already since a long time ago, and it is part of-- we often eat preserved fish or salted fish, very low quality of fish… I also have experience living in Sulawesi or in the eastern part of Indonesia when they only eat high-quality and fresh fish*.

(45-year-old male, an environmental researcher).

Additionally, informants made a direct link between nutrition transition and marine pollution in Indonesia by implying that marine pollution has worsened in Indonesia because of the nutrition transition, through growth in consumerism and reliance on convenience foods, which lead to more volume of organic and industrial waste that get dumped into the sea. Further, they highlighted the higher demand for animal source foods, that led to more animal production plants without adequate drainage system, suggesting further increase in marine pollution from animal waste.

*In the last 20 years, meat consumption is more than doubling. Increase lots ruminant production in the regions around Indonesia. So the lack of drainage system, the food drainage from inland to the ocean, can affect the environment or marine area*.

(47-year-old female, an environmental researcher).

Informants raised concern about the increasing reliance on single-use plastics by the Indonesian society. Many convenience foods come packaged in single-use plastics. The proliferation of online food platforms also means more food orders delivered in this kind of wrapping. Informants further explained that as part of food safety measures, the government promoted the use of single-use plastics and food wrappings, which inadvertently led to a higher volume of plastics that get dumped in the seas and oceans. Informants additionally noted the increase in the use of online food ordering. To further compound these factors, informants explained that control measures to reduce marine pollution by the government are inadequate.

*We are second to China. It means that our waste collection system, or waste management system, it’s not efficient yet … If you go to our coastal area and then you see the kind of plastic waste, mostly are from food-related like powdered drinks, tons of chocolate, margarine, cookies, or biscuit, all packaged in plastic. The worst of all, usually, is also a single pack. Even one biscuit will be wrapped in plastic because of the affordability, because of the income generated by families… Of course, it will create more and more pollution*.

(59-year-old male, an environmental researcher).

*Sometimes, they want to give a punishment to people who are doing harmful activities close to their ocean, but then when the people have more power, like have position in the corporate, there is a lack of law enforcement from the local government*.

(47-year-old female, an environmental researcher).

Informants described other factors that contribute to fish scarcity in the urban centers, including over-fishing and destruction of mangrove growth due to urban development, which leads to destruction of the coral reef through climate change.

*I think what influence the availability is overfishing especially in the coast of Java because we have a lot of fishermen, all of them catch fish, so there’s overfishing. The other one is destruction of our coastal, especially with the destruction of the mangrove, which is important for fish to stay there as part of their reproduction area and nursing area but it’s now getting limited and limited because of developments… but then if there’s no more area … because there’s housing and big water it’s the end of the mangrove. Then also fish availability will be impacted with that expiration*.

(44-year-old male, an environmental researcher).

Informants explained that there is generally a low level of awareness about the impact of marine pollution on fish quality. However, the more people become aware, the more cautious they become about consuming fish.

*I think most of Indonesian, they don’t have enough knowledge. They don’t have enough literacy for the marine pollution ... I think only very particular person who are quite knowledgeable about that and then try to be a bit picky when eating fish*.

(45-year-old female, an environmental researcher).

*Actually, because the common people… they’re just eating as a normal. For example, for other people that they know, for example, the researcher, they did some study checking the population in the fish near the factory, for example. They don’t want to eat that fish from those area. They prefer from other area, for example*.

(38-year-old female, a healthcare provider).

While some informants signaled the need to continue to raise awareness and promote fish consumption, others expressed concern about the unexpected consequences of promoting higher consumption of fish in Indonesia, given the high levels of marine pollution. They emphasized that leaving the problem of plastic pollution unchecked will have very dire impacts on population health in the long term and underscored the urgent need for greater awareness about the impacts of marine pollution.

*This is a paradox. We are promoting very hardly those who live in the coastal area to eat more seafood in order to improve the diet quality, to improve probably the growth of the children and things like that. On the other side of the coin, you have more and more polluted seafood. That’s what happened right now … I would say there is no serious or significant measure to deal with this yet*.

(59-year-old male, an environmental researcher).

*Maybe today, I found a little bit of microplastic, but if you don’t consider, we don’t think, we don’t aware of what is problem, maybe sometime microplastic will be high concentrate in the fish. It can influence our health or next future. That’s why we still campaign about this problem*.

(45-year-old male, an environmental researcher).

## 4. Discussion

By integrating the findings from two different datasets (a population-level cross-sectional survey of fish consumption, and a qualitative survey of key-informants’ perspectives on the impacts of marine pollution on fish quality and availability), we sought to understand the factors related to fish consumption in the larger context of high levels of marine pollution in Indonesia. To our awareness, this is the first study utilizing a mixed-methods approach to investigate the intersectionality of fish consumption and marine pollution in Indonesia.

Our results revealed that overall, fish was the ASF most frequently consumed by survey respondents, but at differing levels based on age-groups. Older respondents (50 years and older) consumed more fish within the week, while younger respondents (15–19 years) consumed slightly more meat (chicken, beef, pork). Key informant interviews results provided a context for this finding by revealing that fish is a relatively cheaper source of animal protein, perceived as beneficial for health. Informants’ perspectives also converged with and extended the survey results by showing that older adults, who consider fish a healthier source of protein than chicken or beef tend to prefer fish more than the younger generation. Previous studies on dietary patterns in Indonesia show similar results. In a previous cross-sectional study on dietary patterns and cardiometabolic risk factors in Indonesia, we reported higher adherence by younger respondents to a modern dietary pattern characterized by more meat, while older respondents reported higher adherence to a traditional pattern that was characterized by more fish [19]. Likewise, we investigated the impacts of the nutrition transition phenomenon on diets and health outcomes of Indonesians using a qualitative method of inquiry, and informants’ responses suggested the same generational divide on food choice [23]. In contrast to our findings, focus group participants in a study of dietary patterns in Indonesia reported similarity on food preferences among the older and younger participants [24]. However, a more recent study by Rachmi and colleagues (2021) upheld our findings [53]. We surmise that the discrepancy could be due to rapid changes in dietary patterns in Indonesia, since the study using focus groups was conducted in 2008, while ours is more recent. We can also attribute the difference to the fact that their study was conducted with respondents from one region of Indonesia (West Sumatra), while our study participants are from different regions [23].

Findings from the quantitative analyses indicated lower fish consumption among urban dwellers, particularly in the Java region. The qualitative results confirm this and the idea that marine pollution negatively impacts fish availability and quality. Available literature from Indonesia echoes this concern. Soegianto et al. reported the presence of heavy metals in clams from East Java Coast at concentration levels considered harmful for human health, including risks for both carcinogenic and non-carcinogenic health effects over the course of lifetime exposure [54]. One study found high levels of zinc above the threshold permitted by the Indonesian government in green mussels from the coastal region of Demak [55]. Candra and colleagues cautioned against consumption of Mantis shrimp from the eastern coast of the Java Sea due to unsafe levels of chloride and lead found in the muscles of this shrimp species [56]. Another study found pathogenic bacterial and viral species in both free-living and aqua-cultured shrimp samples from Bali and Jakarta [57]. An international study comparing microplastics in fish found more microplastics in fish from Indonesia than from two reference cities in China [58].

Our findings on the association of education level with views about fish were more nuanced. Respondents who had a bachelor’s level and above had a higher probability of fish consumption in the quintile 3. Since education is often considered a proxy for socio-economic status (SES), it may well be that respondents with higher education levels are more likely to consume fish moderately because they can afford to buy other ASFs. For respondents in the primary education or less category, we found split probabilities at quintiles 1 and 5 (Figure 3). This could mean that for those at quintile 1, affordability is an issue, while quintile 5 may reflect involvement in fishery, as we found that being involved in fishery occupation is associated with a greater likelihood for fish consumption at the highest quintile (Figure 3 and Supplementary Table S1).

Our results corroborate the few studies from Indonesia that have investigated awareness about marine pollution and its effects on fish desirability. In a study assessing the contribution of fish to the diets and nutrition of women and children in three coastal communities in Indonesia, the authors found that over 50% of mother-child pairs did not meet the minimum recommended dietary diversity, and although fish was the main source of animal-protein in diets, mothers delayed giving fish as complementary foods to infants and young children because of fears of allergies and illnesses [3]. Another study assessed perception of fish quality among Indonesian youths and found that a higher perception of fish quality-assurance scores is correlated with a positive attitude toward and an increase in fish consumption [59]. Wijaya and colleagues used factor analysis to investigate barriers to fish consumption among survey respondents in Yogyakarta and Central Java, reporting that consumer perceptions of fish safety, particularly concerns about contaminations from dangerous chemicals and harmful bacteria, was a major barrier to fish consumption [5]. Our findings add to these studies by suggesting that people’s awareness of marine pollution would influence their desire to consume fish and that the more educated people are, the more likely they will be aware of marine pollution and its impacts, which would then influence their desire to consume fish.

Informants’ views revealed other underlying mechanisms and socio-cultural factors not covered by the cross-sectional survey that may help explain increasing rates of marine pollution, such as increasing rural to urban migration and destruction of the mangrove forests. While previous studies from Indonesia on rural-urban migration have focused on clarifying the classification “urban” [60,61] and on the motives for migration [62,63], to our knowledge, no study has investigated the effects of rural-urban migration on marine pollution. Arifanti (2020) reported that while Indonesia has the largest mangrove coverage in the world, it also has one of the highest rates of mangrove loss [64]. Although the Indonesian government is currently making efforts to restore the mangrove forests, Ilman et al. (2011) reported that the rate of destruction is progressing much more quickly than the rehabilitation efforts [65]. Our findings extend these studies by suggesting that urban-rural migration gives rise to slums and the destruction of the mangrove forests due to housing development in the urban centers, which contribute to marine pollution levels.

This study has limitations. First, for the quantitative analyses, we note the issue of confounding variables that were not captured during the survey, which could have biased the observed associations. Hence, no causal relationship could be inferred from our findings. Also, the instrument for assessing fish consumption in the survey was a brief food screener that was designed to capture specific micronutrients of public health importance for Indonesia and provided no details in quantities of consumption or methods of preparation. This may have introduced measurement errors and confounding, since we were not able to control for total energy intake. Further, survey questions addressed fish consumption only for the previous week; thus, our results may not reflect the habitual fish consumption of respondents. Irrespective of these limitations, assessing fish consumption from a brief food screener is still a valid approach, since food screeners have adequate reproducibility and validity when compared with a longer food frequency questionnaire and biomarkers [66,67].Furthermore, brief food screeners have a low cost and low respondent burden and are appropriate for studies testing a limited set of hypotheses, as in this study [67,68]. Also, our dataset has a large sample size, which allowed us to arrive at more precise and robust estimates of the relationship between fish consumption and all the covariates assessed than a less powered study would have done.

For the qualitative data, most of our key informants are doctoral degree holders. While their perspectives may not accurately represent the perspectives of the Indonesian population with a lower education level, purposively recruiting informants with expertise in the different topic areas of our research was crucial to get expert opinions on the research topics. Future studies may consider using other forms of qualitative approaches such as focus groups and ethnographic studies and recruit participants from the general population to uncover additional perspectives outside the scope of this research. Finally, since the interviews were conducted via Zoom, we understand that in-person interviews may have provided more content in non-verbal cues and facial expressions than we were able to capture in a remote online setting, which tends to be more static. We addressed this as much as possible by keeping the camera on during interviews. Nevertheless, the socio-ecological framework employed to develop the interview questions guide and analyze the themes allowed us to cover a broad scope of topics relating to marine pollution and its impacts on fish availability and quality. Additionally, by recruiting experts from different fields, we gained in-depth insights based on multi-disciplinary perspectives of the different drivers of marine pollution in Indonesia. By combining the two datasets, we were able to get a better understanding of the factors related to fish consumption in the context of marine pollution. The qualitative data expanded and provided contextual meanings for the results of the cross-sectional survey, thus increasing the validity of our results.

### Implications for Policy and Directions for Future Research

Indonesia, an island country with a larger proportion of its surface area made up of ocean than land, is unsurprisingly the world’s second-largest fish producer [12]. However, Indonesia also has one of the highest levels of marine pollution in the world [17]. In 2017, the Indonesian government passed the National Plan of Action (NPOA) on Marine Plastic Debris Management [69]. This five-point action plan was set up to demonstrate the commitment of the Indonesian government to reduce marine plastic litter both at the industrial and domestic levels. Part of the action plan includes campaigns to ban the use of single-use plastics, sort wastes, promote recycling, and set up “waste-banks” in many communities. Irrespective of all these efforts, our informants’ perspectives implied that there has been no discernible improvement in the levels of marine pollution in Indonesia.

Informants identified several challenges to effectively implement waste management policies in Indonesia and proposed some potential solutions. One major barrier to implementing the pollution reduction policies identified was low awareness about the different domestic and industrial factors that contribute to pollution or even how the different wastes get released into the seas and ocean. According to informants, this low awareness exists not just among the general population, but also among some government entities. Another contributory factor highlighted was failure to enforce the different regulations in many communities. Informants also signaled the complexity and high participant burden of some waste management programs. In view of this, informants with expertise in environmental research made the following recommendations:

1. Intensify awareness campaigns in the communities and include participation from universities, non-governmental organizations [70], and other relevant groups from the private sector.
2. Build the capacity of all communities to have adequate facilities to implement the policies at their level.
3. Decentralize the approach to reduce pollution to minimize the burden on communities to organize waste collection.
4. Introduce filtering technology in the rivers and streams so any waste is captured before it gets to the ocean.
5. Put better controls in place to manage wastes and make waste producers pay more for their waste.
6. Cut out the middlemen between the waste banks and the recycling industry, and make it easier for waste banks to get the best deals for selling the collected materials.

Our findings suggest a disconnect between government policy to increase fish consumption and the drive to reduce marine pollution. This could be an indication that the different relevant agencies and sectors are siloed. Even if the government campaign gains traction and people want to eat more fish, their knowledge of marine pollution might keep them from buying fish. They might pivot to other, more expensive protein sources. A good place to start could be for the current campaign to consume more fish to go hand in hand with the awareness of marine pollution. The government might better prioritize making the connection between marine pollution and fish quality and eventually how that impacts the health of the population. The population needs to be educated on how their waste gets into the ocean, how it impacts fish quality and availability, and the adverse impact on their health in the long term. As our results show, corroborated by the relatively few previous studies, higher perception of fish quality affects the desirability of fish consumption. Thus, promoting higher consumption of fish without effectively addressing marine pollution may well turn out to be counterproductive. More studies are needed to better elucidate the challenges related to simultaneously promoting fish consumption and seeking to reduce pollution levels to inform the design of sustainable and cost-effective double-barreled policy solutions.

Marine pollution not only threatens fish quality, but it also negatively impacts fish availability, with potential repercussions on food security and the economy. For instance, an important but cheap protein source may suddenly become non-affordable for low-income families, thereby barring access to an important nutrient source for those who need it most. As we found, fish is an important economic asset for many fisher households, for households can directly consume part of their catch or generate income from the sales of their catch to purchase other food provisions [3]. If marine pollution continues unabated, the livelihood of fish households will be in jeopardy. Further, fish is a main export income generator for Indonesia. Depleting fish catches and lowering fish quality could adversely affect the availability and desirability of Indonesian fish in the international market; this in turn could mean lower GDP earnings from fish export. Future studies should investigate the long-term impact of marine pollution on livelihoods, particularly for fishery households, and on the Indonesian fish export market.

Finally, the effects of marine pollution on human health are not readily evident, but they are gradually emerging with mixed results. Earlier studies reveal a lower negative effect while more recent studies are beginning to reveal concerning trends [54,71,72,73]. Our informants expressed the view that the effect is cumulative and that findings of small effect should not be ignored. Given that Indonesia is the world’s second-largest producer of fish, this may impose dire ramifications on human health on a global scale. Additional studies with longitudinal designs are warranted to track the impact of marine pollution on human health, both for Indonesia and globally.

## 5. Conclusions

The results of both quantitative and qualitative analyses point to a generational divide by age-group for fish preference, with older respondents consuming more than the younger respondents. Qualitative data extend this result by suggesting that food preference among older adults is influenced by health concerns. Furthermore, quantitative analyses revealed relatively lower consumption of fish in urban locations than in rural and in the Java region than in non-Javanese areas. Qualitative results implied that this may be due to depleting fish catches because of marine pollution. Further, our analyses revealed moderate consumption of fish associated with higher educational levels, implying a higher likelihood of substituting fish for other animal-source foods and more nuanced interpretations among those with a lower education level. Our findings suggest that overall, there is a low awareness of the impact of marine pollution on fish quality among Indonesians, but that with increasing awareness, the desire for fish reduces. Moreover, we found that nutritional transition and urbanization are major contributing factors to marine pollution through changing dietary patterns and increasing rates of the depletion of mangrove forests. Finally, our results evoke concern from study informants on the paradox of promoting fish consumption among the Indonesian population while marine pollution levels are rising. While we found that fishery as an occupation was associated with higher fish consumption, the views of key informants indicate that depleting fish catches due to marine pollution may affect not only the food security and livelihoods of fishery households, but also the economy of Indonesia in the long term. More empirical studies, both quantitative and qualitative, are needed to corroborate our findings and inform policies and programs to simultaneously promote fish consumption and mitigate increasing marine pollution levels in Indonesia.

## Figures and Tables

**Table 1:** Characteristics of study respondents in IFLS 5 stratified by age group.

Characteristics	15–19 (*n* = 3550)	20–49 (*n* = 20,876)	≤50 (*n* = 6606)	Total (*n* = 31,032)
**Gender, *n* (%)** ^1^				
Female	1851 (45.6)	11,184 (54.0)	3503 (54.8)	16,538 (53.7)
Male	1699 (54.4)	9692 (46.0)	3103 (45.2)	14,494 (46.3)
**Age, year, mean (sd)**	16.8 (1.8)	35.1 (9.4)	59.6 (6.2)	42.4 (15.8)
**BMI, kg/m^2^ mean (sd)**	20.4 (4.6)	23.8 (4.9)	23.4 (3.5)	23.4(4.6)
**Place of residence, *n* (%)**				
Rural	1372 (48.4)	8449 (49.4)	2932 (52.3)	12,753 (50.3)
Urban	2178 (51.6)	12,427 (50.6)	3674 (47.7)	18,279 (49.7)
**Region**				
Java	1902 (67.8)	11,134 (72.3)	3829 (74.7)	16,865 (72.8)
Kalimantan	164 (3.5)	1096 (3.4)	303 (2.7)	1563 (3.2)
N. Tenggarra	473 (4.7)	2658 (4.8)	771 (4.9)	3902 (4.8)
Sulawesi	181 (3.8)	1050 (3.2)	310 (2.8)	1541 (3.1)
Sumatra	830 (20.2)	4938 (16.4)	1393 (15.0)	7161 (16.1)
**Marital Status, *n* (%)**				
Single	3220 (91.6)	2846 (13.6)	69 (0.9)	6135 (15.0)
Married	319 (8.2)	17,257 (82.1)	4947 (75.1)	22,523(74.0)
Divorced	11 (0.2)	534 (2.8)	245 (3.7)	790 (2.9)
Widowed	0 (0.0)	239 (1.6)	1345 (20.3)	1584 (8.1)
**Education Level, *n* (%)**				
Primary or less	220 (6.7)	5627 (32.7)	4527 (71.9)	10,374 (44.6)
Secondary	3047 (86.8)	11,773 (53.2)	1562 (21.3)	16,382 (44.4)
Some college	66 (1.5)	890 (3.5)	166 (2.2)	1122 (2.9)
Bachelor’s and above	217 (5.1)	2586 (10.7)	351 (4.6)	3154 (8.1)
**Agricultural Occupation**				
Non-Farmer	2435 (66.3)	13,747 (63.1)	3653 (53.1)	19,835 (59.8)
Non-Fish Farmer	1022 (31.4)	6482 (34.6)	2801 (45.3)	10,305 (38.1)
Fishery	93 (2.3)	647 (2.3)	152 (1.6)	892 (2.1)
**Weight Status** ^2^**, *n* (%)**				
Underweight	1124 (32.8)	1891 (9.0)	812 (12.7)	3827 (12.1)
Normal	1779 (50.0)	8336 (38.9)	2491 (38.5)	12,606 (39.6)
Overweight	471 (12.6)	6830 (33.0)	2121 (31.4)	9422 (30.9)
Obese	176 (4.7)	3819 (19.1)	1182 (17.4)	5177 (17.4)

^1^ Percentages are weighted to account for the sampling design and attrition factors. ^2^ Weight status was derived from BMI categories based on Asian population cut-offs.

**Table 2:** Demographic Characteristics of Study Informants.

Characteristics	Total
**Expert Informants**	27
Nutrition/Public Health Researchers	10
Healthcare Providers	8
Environmental Researchers	9
**Age (Year) mean, (**±**SD)**	46.1 (11.9)
**Gender, *n* (%)**	
Female	19 (70.4)
Male	8 (29.6)
**Ethnicity, *n* (%)**	
Javanese	14 (51.9)
Sundanese	2 (7.4)
Batak	2 (7.4)
Buginese	2 (7.4)
Sulawesi	2 (7.4)
Lampungenese	1 (3.7)
Chinese-Indonesian	1 (3.7)
Other	3 (3.11)
**Education Level, *n* (%)**	
Bachelor’s Degree	3 (11.1)
Masters’ Degree	5 (18.5)
Medical Practitioner/Clinical Nutritionist	6 (22.2)
Doctoral Degree (Ph.D)	13 (48.2)
**Years of working experience, *n* (%)**	
Less than 5 years	6 (22.2)
5 to 10 years	3 (11.1)
11 to 20 years	9 (33.3)
More than 20 years	9 (33.3)

**Table 3:** Summary of Informants’ Perspective on Marine Pollution and Fish Consumption.

Theme	Key Findings
Importance of fish to Indonesian diets	Awareness that fish is an affordable source of protein for many people High awareness about the health and nutritional benefits of fish Differential preference for choosing fish over other animal source foods by age group
Factors related to marine pollution and impacts on fish availability and quality	Increasing rural to urban migration promotes the development of slums Growing consumerism Increasing industrial wastes Overfishing Destruction of mangrove growth due to urban development Higher demand for other animal-source foods, leading to more animal feeding lots Proliferation of plastic packaging industries to meet increasing demand for single-use plastics Reduced fish catches in some urban centers Low quality of fish
Awareness about marine pollution and impact on fish desirability	General low level of awareness about the impact of marine pollution on fish quality The more awareness of the impact of marine pollution, the more caution about fish consumption Growing concerns about promoting fish consumption because of increasing levels of marine pollution

**Table 4:** Mean Consumption of animal source foods (ASF) in IFLS5 by age-group.

ASF	Mean	±SD	25 pctl	50 pctl	75 pctl	Min	Max
**Fish (*n* = 31,032)**	2.8	2.6	1.0	2.0	5.0	0.0	7.0
15–19 year (*n* = 3550)	2.4	2.9	1.0	2.0	4.0	0.0	7.0
20–49 year (*n* = 20,876)	2.8	2.7	1.0	2.0	5.0	0.0	7.0
≥50 year (*n* = 6606)	2.9	2.1	1.0	2.0	7.0	0.0	7.0
**Meat (*n* = 31,032)**	1.5	1.8	0.0	1.0	2.0	0.0	7.0
15–19 year (*n* = 3550)	1.6	2.2	0.0	1.0	2.0	0.0	7.0
20–49 year (*n* = 20,876)	1.5	1.9	0.0	1.0	2.0	0.0	7.0
≥50 year (*n* = 6606)	1.3	1.4	0.0	1.0	2.0	0.0	7.0
**Eggs (*n* = 31,032)**	2.4	2.1	1.0	2.0	3.0	0.0	7.0
15–19 year (*n* = 3550)	2.5	2.4	1.0	2.0	3.0	0.0	7.0
20–49 year (*n* = 20,876)	2.6	2.3	1.0	2.0	3.0	0.0	7.0
≥50 year (*n* = 6606)	2.2	1.6	0.0	2.0	3.0	0.0	7.0
**Dairy (*n* = 31,032)**	1.1	2.2	0.0	0.0	1.0	0.0	7.0
15–19 year (*n* = 3550)	1.4	2.8	0.0	0.0	2.0	0.0	7.0
20–49 year (*n* = 20,876)	1.1	2.3	0.0	0.0	1.0	0.0	7.0
≥50 year (*n* = 6606)	1.1	1.7	0.0	0.0	1.0	0.0	7.0

**Table 5:** Fish Consumption Quintiles by Sociodemographic Characteristics.

Characteristics	Quintiles of Fish Consumption						
	Quintile 1 (*n* = 6104)	Quintile 2 (*n* = 4948)	Quintile 3 (*n* = 7449)	Quintile 4 (*n* = 4909)	Quintile 5 (*n* = 7622)	Total (*n* = 31,032)	*p*-Value ^1^
**Age, years, mean (SD)**	43.0 (16.4)	42.0 (15.0)	41.9 (15.1)	39.9 (16.0)	44.3 (15.9)	42.4 (15.8)	<0.01
**BMI, kg/m^2^, mean (SD)**	23.0 (4.3)	23.6 (4.6)	23.1 (4.3)	23.8 (4.9)	23.6 (4.9)	23.9 (4.6)	<0.01
**Age-group, *n* (%)** ^2^							<0.01
15–19	882 (9.3)	573 (7.3)	650 (5.8)	744 (10.5)	701 (5.9)	3550 (7.6)	
20–49	3819 (52.9)	3359 (58.7)	5294 (60.3)	3379 (60.9)	5025 (54.2)	20,876 (57.1)	
50 and older	1403 (37.8)	1016 (34.0)	1505 (33.9)	786 (28.6)	1896 (39.9)	6606 (35.3)	
**Gender, *n* (%)**							<0.01
Female	3362 (55.4)	2719 (55.2)	3027 (40.6)	3424 (70.4)	4006 (54.3)	16,538 (53.7)	
Male	2742 (44.6)	2229 (44.8)	4422 (59.4)	1485 (29.6)	3616 (45.7)	14,494 (46.3)	
**Place of Residence, *n* (%)**							<0.01
Rural	2448 (50.8)	1812 (45.0)	2793 (47.3)	1845 (48.9)	3855 (58.1)	12,753 (50.3)	
Urban	3656 (49.2)	3136 (55.0)	4656 (52.7)	3064 (51.1)	3767 (41.9)	18,279 (49.7)	
**Region, *n* (%)**							<0.01
Java	4584 (86.5)	3156 (79.4)	4370 (75.9)	2417 (68.9)	2338 (53.0)	16,865 (72.8)	
Kalimantan	112 (1.0)	103 (1.2)	269 (2.2)	303 (4.0)	776 (7.4)	1563 (3.2)	
N. Tenggarra	503 (2.8)	551 (3.9)	811 (3.9)	577 (4.7)	1460 (8.6)	3902 (4.8)	
Sulawesi	61 (0.5)	50 (0.6)	129 (1.0)	172 (1.8)	1129 (10.9)	1541 (3.1)	
Sumatra	844 (9.2)	1088 (14.9)	1870 (17.0)	1440 (20.5)	1919 (20.2)	7161 (16.1)	
**Marital Status, *n* (%)**	Start here						<0.01
Single	1433 (17.4)	958 (14.7)	1420 (15.2)	1127 (17.3)	1197 (11.1)	6135 (15.0)	
Married	4099 (69.0)	3627 (74.6)	5562 (75.9)	3424 (71.7)	5811 (78.1)	22,523 (74.0)	
Divorced	171 (3.0)	114 (2.6)	168 (2.7)	117 (2.8)	220 (3.2)	790 (2.9)	
Widowed	401 (10.6)	249 (8.1)	299 (6.1)	241 (8.1)	394 (7.6)	1584 (8.1)	
**Education Level, *n* (%)**							<0.01
Primary or less	2296 (49.5)	1661 (43.8)	2237 (40.1)	1304 (38.3)	2876 (49.3)	10,374 (44.6)	
Secondary	3216 (42.9)	2727 (46.5)	4038 (46.5)	2717 (47.8)	3684 (40.0)	16,382 (44.4)	
Some college	171 (2.3)	165 (2.8)	305 (3.5)	252 (3.8)	229 (2.4)	1122 (2.9)	
Bachelor’s and over	421 (5.3)	395 (6.9)	869 (10.0)	636 (10.2)	833 (8.3)	3154 (8.1)	
**Agricultural Occupation**							
Non-Farmer	4100 (61.7)	3369 (64.7)	4942 (61.4)	3296 (61.9)	4128 (51.1)	19,835 (59.8)	
Non-Fish Farmer	1940 (37.5)	1527 (34.4)	2386 (37.4)	1479 (36.0)	2973 (43.8)	10,305 (38.1)	
Fishery	64 (0.8)	52 (0.9)	121 (1.2)	134 (2.2)	521 (5.1)	892 (2.1)	
**Weight Status** ^3^**, *n* (%)**							<0.01
Underweight	883 (14.0)	613 (11.6)	905 (12.1)	593 (11.3)	833 (11.3)	3827 (12.1)	
Normal	2596 (41.7)	1973 (38.7)	3071 (40.9)	1875 (36.1)	3091 (39.1)	12,606 (39.6)	
Overweight	1703 (28.8)	1454 (30.3)	2315 (31.3)	1562 (33.2)	2388 (31.5)	9422 (30.9)	
Obese	922 (15.6)	908 (19.5)	1158 (15.8)	879 (19.5)	1310 (18.1)	5177 (17.4)	

^1^ *p*-values based on Pearson’s chi-square tests for categorical variables and ANOVA for continuous variables. ^2^ Percentages are weighted to account for sampling design and attrition factor. ^3^ Weight status derived from BMI categories based on Asian population cut-offs.

## Data Availability

Data sharing is not applicable to this article.

## Supplementary Materials

The following supporting information can be downloaded at: https://www.mdpi.com/article/10.3390/ijerph20085582/s1, Table S1: Multinomial regression analyses of the association between quintiles of fish consumption and sociodemographic factors.

## Abbreviations

ASFs	Animal source foods
BMI	Body mass index
EAs	Enumeration Areas
IFLS	Indonesian Family Life Survey
IRB	Institutional Review Board
LMICs	Lower-middle income countries
PUFAs	Poly-unsaturated fatty acids
RAND	Research and Development Corporation
SEM	Socio-ecological model
UNDIP	Universitas Diponegoro
WHO	World Health Organization

## Appendix A. Interview Question Guide

Impacts of marine pollution on fish quality and availability

1. Now, let’s consider the impacts of marine pollution:
  1.1 To your knowledge, what are some of the major sources of marine pollution?
  1.2 Which regions of Indonesia are particularly liable to marine pollution? Why?
  1.3 What are your thoughts about the contribution of fish to the diet of Indonesians?
  1.4 In what ways, if any, does marine pollution impact fish quality?
  1.5 What about fish availability?
  1.6 In what ways, if any, has fish quality impacted the importance given to fish consumption in the Indonesian diet?
  1.7 In what ways, if any, does marine pollution affect the availability and quality of other healthy food types such as fruits and vegetables, whole grains, and nuts?
2. Now, I want us to discuss the relationship between nutrition transition and marine pollution:
  2.1 Indonesia ranks second globally after China for man-made marine pollution. In your opinion, what factors contribute to this?
  2.2 Do you think nutrition transition is related to marine pollution?
    2.2.1 If yes, how so?
    2.2.2 In what ways, if any, has the westernization of the food system contributed to marine pollution? (If not already addressed in 2.2 above.)
3. Other factors
  3.1 What other environmental factors threaten fish quality and availability?
    Probe: Are there other types of pollution (apart from marine pollution), that threaten fish quality?
    3.1.1 What about fish availability?
  3.2 What about availability and quality of other healthy food types such as fruits, vegetables, whole grains, and nuts?
4. Now, let’s discuss government policy on reducing pollution:
  4.1 Tell me about the current policies in place to reduce marine pollution in Indonesia that you’re aware of.
  4.2 What major barriers have there been, if any, to implementing these policies?
  4.3 In your opinion, what can the government do differently?
  4.4 What other factors beyond nutrition comes to mind, that might have adverse effects on the health of Indonesians, that we have not touched upon?
