# Supplementary material for: Fish—To Eat or Not to Eat? A Mixed-Methods Investigation of the Conundrum of Fish Consumption in the Context of Marine Pollution in Indonesia
Source: Int J Environ Res Public Health. 2023 Apr 19;20(8):5582. doi: 10.3390/ijerph20085582 (PMC10138686; doi:10.3390/ijerph20085582)
Supplement: Supplementary file 1 [file ijerph-20-05582-s001.zip › ijerph-2161859-supplementary.pdf]

Supplemental Table S1: Multinomial regression analyses of the association between quintiles[1] of fish consumption and sociodemographic factors

	**Quintile 2[1] (n = 4,948)**				**Quintile 3[1] (n = 7,449)**				**Quintile 4[1] (n = 4,909)**				**Quintile 5[1] (n = 7,622)**			
**Characteristics**	**Adj. RRR[3]**	**P-val**	**95% CI LL**	**95% CI UL**	**Adj. RRR[3]**	**P-val**	**95% CI LL**	**95% CI UL**	**Adj. RRR[3]**	**P-val**	**95% CI LL**	**95% CI UL**	**Adj. RRR[3]**	**P-val**	**95% CI LL**	**95% CI UL**
**Education Level, n (%)[2]**																
Primary or less 10,374 (44.6)	1.00	-	-		1.00	-	-		1.00	-		-	1.00	-	-	-
Secondary 16,382 (44.4)	1.14	0.020	1.02	1.27	1.17	0.002	1.06	1.29	1.21	0.002	1.07	1.36	0.97	0.528	0.87	1.07
Some college 1,122 (2.9)	1.21	0.162	0.93	1.58	1.51	0.001	1.19	1.91	1.58	0.000	1.23	2.02	1.00	0.984	0.76	1.30
≥ Bachelors 3,154 (8.1)	1.30	0.007	1.07	1.57	1.84	0.000	1.56	2.18	1.90	0.000	1.58	2.29	1.49	0.000	1.25	1.78
**Agricultural Occupation, n (%)[2]**																
Non-farmer 19,835 (59.8)	1.00	-	-	-	1.00	-	-	-	1.00	-	-	-	1.00	-	-	-
Non-fish farmer 10,305 (38.1)	0.96	0.477	0.86	1.07	1.07	0.176	0.97	1.19	1.06	0.360	0.94	1.18	1.19	0.002	1.07	1.32
Fishery occupation 892 (2.1)	1.02	0.941	0.66	1.57	1.47	0.038	1.02	2.12	2.70	0.000	1.87	3.89	5.65	0.000	4.10	7.79
**Age Group, n (%)[2]**																
15 - 19 Y 3,550 (7.6)	0.89	0.190	0.75	1.06	0.66	0.000	0.56	0.78	1.24	0.016	1.04	1.48	1.01	0.927	0.84	1.21
20 - 49 Y 20,876 (57.1)	1.00	-	-	-	1.00	-	-	-	1.00	-	-	-	1.00	-	-	-
≥ 50 Y 6,606 (35.3)	0.88	0.039	0.78	0.99	0.93	0.181	0.83	1.04	0.80	0.001	0.70	0.92	1.18	0.004	1.05	1.33
**Gender, n (%)[2]**																
Male 14,494 (46.3)	1.00	-	-	-	1.00	-	-	-	1.00	-	-	-	1.00	-	-	-
Female 16,538 (53.7)	0.96	0.429	0.88	1.06	0.56	0.000	0.51	0.61	2.03	0.000	1.83	2.24	0.91	0.050	0.83	1.00
**Place of Residence, n (%)[2]**																
Rural 16,538 (53.7)	1.00	-	-	-	1.00	-	-	-	1.00	-	-	-	1.00	-	-	-
Urban 18,279 (49.7)	1.22	0.000	1.10	1.36	1.09	0.081	0.99	1.20	0.98	0.676	0.88	1.09	0.85	0.002	0.76	0.94
**Region, n (%)[2]**																
Java 16,865 (72.8)	1.00	-	-	-	1.00	-	-	-	1.00	-	-	-	1.00	-	-	-
Kalimantan 1,563 (3.2)	1.41	0.028	1.04	1.91	2.52	0.000	1.96	3.25	4.80	0.000	3.73	6.19	11.16	0.000	8.85	14.06
N. Tengarra 3,902 (4.8)	1.54	0.000	1.33	1.79	1.64	0.000	1.43	1.88	2.09	0.000	1.79	2.43	4.96	0.000	4.35	5.66
Sulawesi 1,541 (3.1)	1.36	0.150	0.90	2.05	2.51	0.000	1.78	3.55	5.03	0.000	3.60	7.01	40.37	0.000	30.11	54.12
Sumatra 7,161 (16.1)	1.81	0.000	1.61	2.04	2.16	0.000	1.95	2.41	2.90	0.000	2.59	3.26	3.55	0.000	3.18	3.97
**Marital Status, n (%)[2]**																
Divorced 790 (2.9)	1.16	0.359	0.85	1.59	1.41	0.019	1.06	1.89	1.25	0.189	0.90	1.74	2.00	0.000	1.46	2.74

Married 22,523 (74.0)	1.37	0.000	1.17	1.59	1.40	0.000	1.22	1.60	1.31	0.001	1.12	1.53	1.95	0.000	1.67	2.29
Single 6,135 (15.0)	1.00	-	-	-	1.00	-	-	-	1.00	-	-	-	1.00	-	-	-
Widowed 1,584 (8.1)	1.09	0.498	0.85	1.40	1.05	0.665	0.83	1.33	1.06	0.665	0.81	1.38	1.28	0.056	0.99	1.64
**Other Animal Source Foods**																
Meat (beef, chicken, pork)	1.02	0.097	1.00	1.06	1.06	0.000	1.03	1.09	1.14	0.000	1.11	1.17	1.08	0.000	1.05	1.11
Eggs	0.97	0.024	0.95	1.00	1.07	0.000	1.05	1.09	1.09	0.000	1.07	1.12	1.10	0.000	1.07	1.13
Dairy	1.00	0.998	0.98	1.02	1.03	0.002	1.01	1.05	1.05	0.000	1.03	1.07	1.06	0.000	1.04	1.08
**Weight Status n (%)[4]**																
Normal Weight 12,606 (39.6)	1.00	-	-	-	1.00	-	-	-	1.00	-	-	-	1.00	-	-	-
Obese 5,177 (17.4)	1.26	0.001	1.10	1.44	1.11	0.094	0.98	1.26	1.16	0.035	1.01	1.34	1.27	0.001	1.11	1.44
Overweight 9,422 (30.9)	1.08	0.173	0.97	1.21	1.12	0.027	1.01	1.24	1.17	0.009	1.04	1.31	1.13	0.023	1.02	1.27
Underweight 3,827 (12.1)	0.96	0.556	0.83	1.11	0.97	0.694	0.85	1.11	1.00	0.976	0.85	1.16	0.93	0.366	0.80	1.09
Constant	0.47	0.000	0.38	0.57	0.62	0.000	0.51	0.74	0.14	0.000	0.11	0.17	0.21	0.000	0.17	0.26

1. Base outcome = Quintile 1.
2. N is weighted to account for sampling design and attrition factor.
3. Adjusted RRR= Relative Risk Ratio; sociodemographic variables: education level, age, gender, place of residence, region, marital status, agricultural occupation simultaneously included in the model; additionally adjusted for weight status, other ASFs: meat, eggs, dairy
4. Weight status derived from BMI categories based on Asian population cut-offs.
